# Stroke-related sarcopenia: a scoping review of influencing factors and clinical outcomes

**DOI:** 10.3389/fragi.2025.1658943

**Published:** 2025-11-06

**Authors:** Hongyan Yang, Ting Yang, Hui Wei

**Affiliations:** Department of Nursing, The Second Affiliated Hospital Zhejiang University School of Medicine, Hangzhou, China

**Keywords:** stroke, sarcopenia, influencing factors, outcomes, scoping review

## Abstract

**Background:**

Stroke-related sarcopenia has attracted increasing attention, and the prevalence is increasing. However, the influencing factors and clinical outcomes are still not well reported in the literature, and existing studies are heterogeneous in terms of study design, outcomes, and means of outcome assessment. We conducted this scoping review to map and summarize the evidence in the rapidly growing field of stroke-related sarcopenia, and guide future research directions.

**Purpose:**

To synthesize the influencing factors and clinical outcomes of stroke-related sarcopenia.

**Methods:**

The scoping review process followed the methodological framework of Arksey and O’Malley and was reported using the PRISMA-ScR guideline. Six English databases (PubMed, Embase, CINAHL, Scopus, Web of Science, and the Cochrane Library) were searched from the inception to 13 August 2024, and updated on 5 October 2025. We included studies involving influencing factors and clinical outcomes (concept) of stroke-related sarcopenia (population) in any setting (context).

**Results:**

Twenty-six studies were identified, including six cross-sectional and twenty cohort studies. Forty influencing factors were extracted and integrated into five categories, including demographic, disease, stroke-related, behavioral, and biomarker factors. Stroke-related sarcopenia can cause impaired motor, swallowing, neurological, and psychological function and lead to increased recurrence, readmission, and mortality.

**Conclusion:**

Our scoping review shows that stroke-related sarcopenia depends on multiple factors and has widespread effects. Understanding these influencing factors and clinical outcomes can help health professionals to intervene and manage stroke-related sarcopenia. However, heterogeneity in the details of the included studies made it difficult to undertake quantitative summaries across studies, more high-quality, multicenter studies should be conducted in the future to provide consistent evidence to guide clinical practice.

## 1 Introduction

Sarcopenia is an age-related geriatric syndrome characterized by loss of muscle mass and decline in muscle strength and function (Cruz-Jentoft and Sayer, 2019), and it is associated with a higher risk of adverse health outcomes, including falls, physical dysfunction, frailty, and increased mortality (Kitamura et al., 2021). Sarcopenia is usually divided into primary and secondary types. Sarcopenia caused by aging is called primary sarcopenia, which is a manifestation of the aging process of the body and is common in the elderly population (Bauer et al., 2019). In addition, activity-related, disease-related, and nutrition-related sarcopenia have been proposed as secondary sarcopenia, and disease-related can accelerate the progression of muscle atrophy and become a part of the disease process (Li et al., 2020). Cancer-related sarcopenia and diabetes mellitus-related sarcopenia have been reported in previous studies (Bozzetti, 2024; Liu et al., 2024). In recent years, stroke-related sarcopenia has attracted increasing attention.

Stroke is the leading cause of death and disability worldwide (GBD, 2019 Diseases and Injuries Collaborators, 2020). Half of stroke survivors are left disabled, with a third relying on others to assist with activities of daily living (Markus, 2022). After stroke, muscle structural changes can be observed shortly, characterized by the loss of motoneurons, atrophy, adjacent reinnervation, and fiber type shift contrasting that of normal aging (Scherbakov et al., 2015). Li et al. (2020) stated that stroke-related sarcopenia can promote the occurrence and development of sarcopenia through a variety of pathogenesis, such as immobilization, impaired feeding, sympathetic activation, inflammation, and denervation. Though the specific mechanism of stroke-related sarcopenia is still unclear, it has obvious characteristics, such as rapid decline in muscle mass (unrelated to aging), structural changes in muscles (transfer of muscle fibers to rapidly contracting fibers), brain damage that determines differences in bilateral physical performance, catabolic signal activation of neurotrophic imbalance (Markus, 2022).

A recent meta-analysis has shown that the prevalence of stroke-related sarcopenia ranges from 16.8% to 60.3%, with a total prevalence of 42% (Su et al., 2020). Inoue et al. (2022) reported that the prevalences of sarcopenia within 10 days of stroke, and from 10 days to 1 month after stroke were 29.5% and 51.6%, respectively. The prevalence has significantly increased, indicating that the intervention for sarcopenia in stroke patients is relatively low, and the clinical outcomes of stroke-related sarcopenia have not received sufficient attention. Stroke-related sarcopenia can be promoted to postpone or prevent negative health outcomes by focusing on influencing factors. Early original studies have explored various influencing factors that affect stroke-related sarcopenia, including age, body mass index (BMI), smoking history, malnutrition, ability of walking, albumin, and so on (Ikeji et al., 2023; Mohammed and Li, 2022; Yao et al., 2022). So describing and mapping influencing factors that affect stroke-related sarcopenia become of paramount importance. In addition, there is still a discrepancy and uncertainty regarding the influencing factors associated with stroke-related sarcopenia. According to some studies, people with stroke history were more likely to experience stroke-related sarcopenia (Yoshimura et al., 2018; Wong et al., 2022). Several studies, however, found no significant relationship between stroke history and stroke-related sarcopenia (Ikeji et al., 2023; Yoshimura et al., 2021). Therefore, an evidence-based review is required.

Sarcopenia is a known risk factor for poor functional outcomes in patients with vascular disease or metabolic syndrome (Pizzimenti et al., 2020; Nishikawa et al., 2021), but the clinical outcomes of stroke-related sarcopenia are still unclear. Some studies have shown that stroke-related sarcopenia is an important predictor of poor functional outcomes (Yoshimura et al., 2019; Kanai et al., 2022). But Matsushita et al. (2019) reported that sarcopenia was significantly associated with functional outcomes at discharge for men, but not for women. In addition, Nishioka et al. (2022) revealed that stroke-related sarcopenia was independently associated with poor swallowing outcomes while Kanai et al. (2022) failed to detect the relationship between stroke-related sarcopenia and swallowing outcomes. The existing literature on clinical outcomes of stroke-related sarcopenia is heterogeneous and inconclusive, so clarifying the impact of sarcopenia in stroke patients may have important clinical implications. Patients with stroke-related sarcopenia are more likely to experience adverse outcomes such as depression, readmission, and death (Shiraishi et al., 2024; Abe et al., 2024; Tutal et al., 2023). Therefore, it is intuitive to assume that promoting stroke-related sarcopenia can potentially prevent adverse events.

In the field of stroke, influencing factors related to sarcopenia and the impacts of sarcopenia on patients are still not well reported in the literature, and existing studies are heterogeneous in terms of study design, outcomes, and means of outcome assessment. Scoping review, is a method for synthesizing research evidence, and is used for classifying the main elements in a field or identifying gaps in the existing literature (Pham et al., 2014). Thus, we conducted this scoping review to map and summarize the evidence in the rapidly growing field of stroke-related sarcopenia, focusing on the influencing factors and clinical outcomes of stroke-related sarcopenia, and to guide future research directions.

## 2 Methods

This scoping review process followed the methodological framework of Arksey and O’Malley (Arksey and O'Malley, 2005), including identifying the research question, identifying relevant studies, selecting studies, charting the data, and collating, summarising, and reporting the results. The Preferred Reporting Items for Systematic Reviews and Meta-Analyses extension for Scoping Reviews (PRISMA-ScR) (Tricco et al., 2018) was used to optimize reporting. PRISMA-ScR checklist is presented in Supplementary Appendix 1. This scoping review was not registered.

### 2.1 Identifying the research question

The specific research questions that guided this scoping review were as follows:

- What influencing factors affect stroke-related sarcopenia?
- What are the clinical outcomes of stroke-related sarcopenia?

### 2.2 Identifying relevant studies

Six electronic databases, including PubMed, Embase, CINAHL, Scopus, Web of Science, and the Cochrane Library were used for this review. All databases were searched from the inception to 13 August 2024, and updated on 5 October 2025. The reference lists in the included studies were traced back to identify additional studies. Our research team consulted 2 information specialists and reviewed previous relevant studies to develop search strategies. The search strategy combined terms for (1) stroke, and (2) sarcopenia, and has been included as Supplementary Appendix 2. The PCC (Population/Concept/Context) framework is recommended by JBI to identify eligible criteria (Peters et al., 2021).

Inclusion criteria.

- Population: this review considered all studies focusing on stroke-related sarcopenia. And population was diagnosed with stroke by any available diagnostic criteria.
- Concept: studies involving the influencing factors and clinical outcomes of stroke-related sarcopenia.
- Context: any clinical context (all countries and healthcare settings, e.g., acute care, primary healthcare, and community setting).

Exclusion criteria.

- Studies that diagnostic criteria of sarcopenia were not clearly reported.
- Non-observational studies.
- The language of the publication was not English.
- Newspaper articles, comments, and conference abstracts.

### 2.3 Selecting studies

All identified citations were exported to EndNote X9. After deleting duplicate articles, the study selection was conducted in two steps. Two investigators independently reviewed the titles and abstracts against the inclusion and exclusion criteria in the first step. In the second step, The full text of potentially relevant studies was screened against the eligibility criteria. Any disagreements were resolved by consensus with a third review investigator.

### 2.4 Charting the data

Our research team developed a standardized data extraction table. Two investigators independently extracted following data: author, year of publication, country, study design, sample, population, setting, diagnostic criteria of sarcopenia, sarcopenia prevalence, influencing factors, and clinical outcomes of stroke-related sarcopenia. In case of disagreements, a third investigator was involved.

### 2.5 Collating, summarising and reporting the results

Data information from the articles was reviewed, summarised, and reported as the study findings. We created the table and figure summarising and describing the influencing factors and clinical outcomes of stroke-related sarcopenia. Disagreements in the article selection between two authors were resolved through discussion by involving the third author until a consensus was reached.

## 3 Results

### 3.1 Overview of selected papers

**FIGURE 1:**
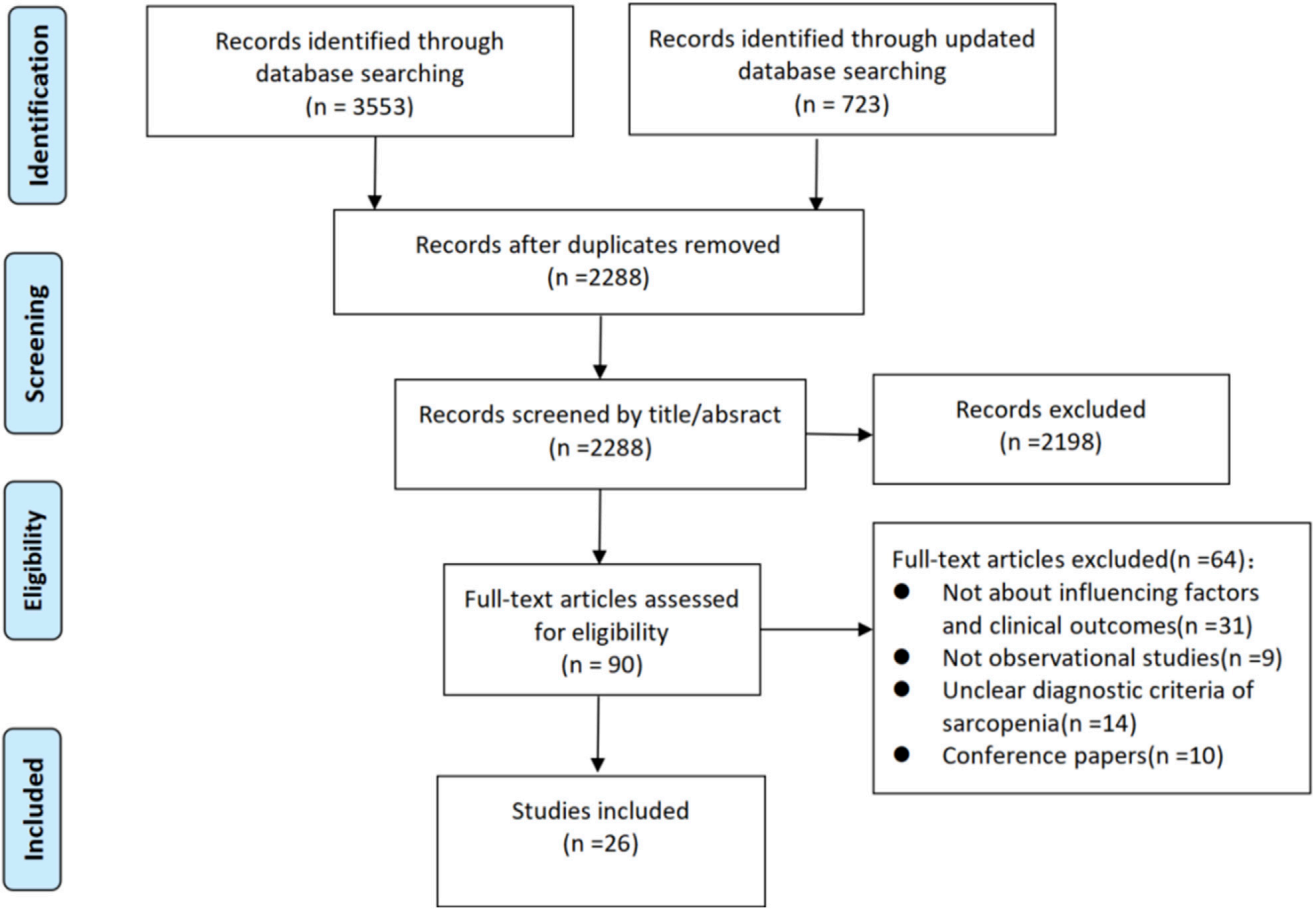
Flow chart of study selection process.

The six electronic databases and references screening yielded 4,276 studies. We removed 1988 duplicates, leaving 2,288 studies. Of these, 2,198 were excluded through the title and abstract screening process, and 90 were reminded for full-text screening. 64 studies were excluded with reasons: not about influencing factors and clinical outcomes (n = 21); not observational studies (n = 9); unclear diagnostic criteria of sarcopenia (n = 14); conference papers (n = 10). Ultimately 26 studies (Ikeji et al., 2023; Mohammed and Li, 2022; Yao et al., 2022; Yoshimura et al., 2018; Wong et al., 2022; Yoshimura et al., 2021; Yoshimura et al., 2019; Kanai et al., 2022; Matsushita et al., 2019; Nishioka et al., 2022; Shiraishi et al., 2024; Abe et al., 2024; Tutal et al., 2023; Aydin et al., 2021; Kim and Choi, 2023; Kim et al., 2025; Shimizu et al., 2022; Yoshimura et al., 2020; Abe et al., 2023; Jang et al., 2020; Kameyama et al., 2022; Lee et al., 2022; Lee et al., 2023; Nozoe et al., 2024; Ogino et al., 2024; Kirkham et al., 2023) were included in our review. A flow chart of the study selection is presented in Figure 1.

### 3.2 Design characteristics

The included studies were published from 2018 to 2024. The sample size varied from 80 to 813 participants. As for study design, six cross-sectional studies, six prospective cohort studies, and fourteen retrospective cohort studies. Fifteen studies were conducted in Japan, five in Korea, two in Turkey, one in China, England, and Malaysia respectively, and one multicenter study in Egypt and China. Among these studies, eighteen used skeletal muscle mass index (SMI) and hand grip strength (HGS) to diagnose sarcopenia, three studies used strength, assistance walking, rising from a chair, climbing stairs and falls (SARC-F), three used calf circumference (CC) and HGS, one used HGS and one used SMI. The basic characteristics of the included studies are shown in Supplementary Table S1.

### 3.3 Influencing factors for stroke-related sarcopenia

#### 3.3.1 Demographic factors

Ten studies (Ikeji et al., 2023; Mohammed and Li, 2022; Yao et al., 2022; Yoshimura et al., 2018; Yoshimura et al., 2021; Aydin et al., 2021; Kim and Choi, 2023; Kim et al., 2025; Shimizu et al., 2022; Yoshimura et al., 2020) looked at the effect of age on stroke-related sarcopenia, with nine studies (Ikeji et al., 2023; Mohammed and Li, 2022; Yao et al., 2022; Yoshimura et al., 2018; Yoshimura et al., 2021; Aydin et al., 2021; Kim and Choi, 2023; Shimizu et al., 2022; Yoshimura et al., 2020) discovering a significant association between age and stroke-related sarcopenia, indicating that older adults were likely to have stroke-related sarcopenia. Only one study (Kim et al., 2025) showed no differences between age and stroke-related sarcopenia.

Seven studies (Ikeji et al., 2023; Yoshimura et al., 2018; Yoshimura et al., 2021; Kim and Choi, 2023; Kim et al., 2025; Shimizu et al., 2022; Yoshimura et al., 2020) explored an association between sex and stroke-related sarcopenia. All studies revealed that women were more likely to have stroke-related sarcopenia.

Five studies (Ikeji et al., 2023; Yoshimura et al., 2018; Wong et al., 2022; Kim and Choi, 2023; Kim et al., 2025) investigated the relationship between BMI and stroke-related sarcopenia. Three studies (Ikeji et al., 2023; Yoshimura et al., 2018; Wong et al., 2022) revealed that participants with a higher BMI were more likely to have stroke-related sarcopenia. However, the other two studies (Kim and Choi, 2023; Kim et al., 2025) failed to identify the association between BMI and stroke-related sarcopenia.

**FIGURE 2:**
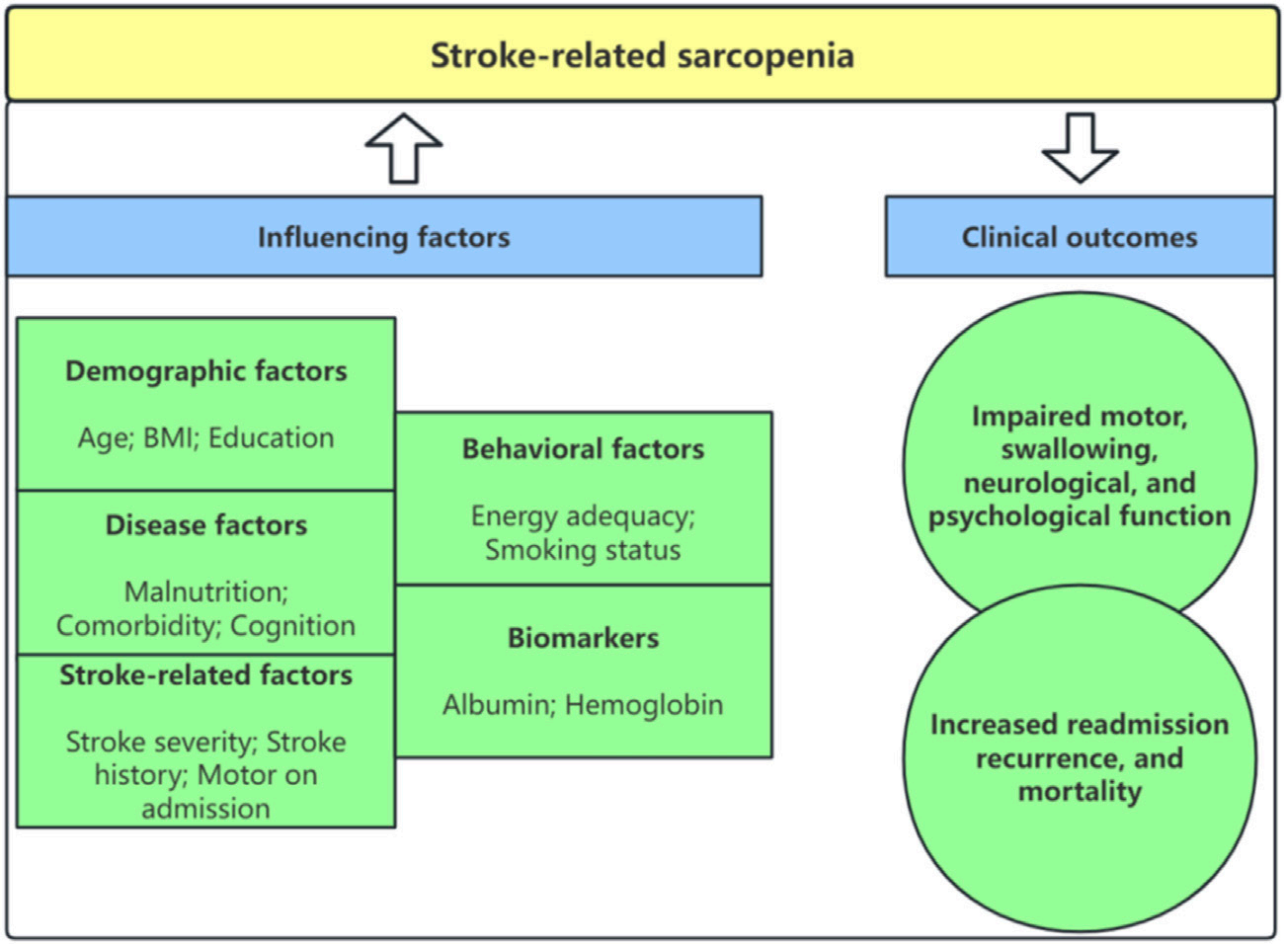
The model of influencing factors and clinical outcomes of stroke-related sarcopenia.

The correlation between education and stroke-related sarcopenia was investigated in two studies (Yao et al., 2022; Kim and Choi, 2023), and it was discovered that education had a significant impact on stroke-related sarcopenia. The weight was reported by only one study (Yao et al., 2022), indicating that weight was relevant to stroke-related sarcopenia. The details are shown in Supplementary Table S2 and Figure 2.

#### 3.3.2 Disease factors

Five studies (Ikeji et al., 2023; Mohammed and Li, 2022; Yoshimura et al., 2018; Wong et al., 2022; Yoshimura et al., 2021) explored the association between malnutrition risk and stroke-related sarcopenia. Three studies (Mohammed and Li, 2022; Wong et al., 2022; Yoshimura et al., 2021) revealed that participants with malnutrition risk were more likely to have stroke-related sarcopenia. However, the other two studies (Ikeji et al., 2023; Yoshimura et al., 2018) failed to identify the association between malnutrition risk and stroke-related sarcopenia.

Four studies (Ikeji et al., 2023; Yoshimura et al., 2018; Yoshimura et al., 2021; Yoshimura et al., 2020) examined the relationship between the length of hospital and stroke-related sarcopenia and showed no differences between length of hospital and stroke-related sarcopenia.

Three studies (Yoshimura et al., 2021; Kim and Choi, 2023; Yoshimura et al., 2020) explored the connection between comorbidity and stroke-related sarcopenia. Two studies (Yoshimura et al., 2021; Kim and Choi, 2023) demonstrated that there were no differences between comorbidity and stroke-related sarcopenia. And only one study (Yoshimura et al., 2020) found that comorbidity was a risk factor for stroke-related sarcopenia.

Two studies (Mohammed and Li, 2022; Yao et al., 2022) examined the relationship between cognition and stroke-related sarcopenia, indicating that cognitive impairment was relevant to stroke-related sarcopenia. The other eight relevant factors were reported by only one study.

#### 3.3.3 Stroke-related factors

Six studies (Ikeji et al., 2023; Mohammed and Li, 2022; Yao et al., 2022; Yoshimura et al., 2021; Kim and Choi, 2023; Kim et al., 2025) investigated the relationship between stroke severity and stroke-related sarcopenia. And 2 studies (Mohammed and Li, 2022; Yao et al., 2022) discovered severe stroke severity was a risk factor for stroke-related sarcopenia. However, no relationship was found in the other four studies (Ikeji et al., 2023; Yoshimura et al., 2021; Kim and Choi, 2023; Kim et al., 2025).

The correlation between stroke history and stroke-related sarcopenia was investigated in four studies (Ikeji et al., 2023; Yoshimura et al., 2018; Wong et al., 2022; Yoshimura et al., 2021). Only one study (Yoshimura et al., 2018) found a significant association between stroke history and stroke-related sarcopenia, with recurrent stroke being more likely to have stroke-related sarcopenia. However, the other three studies (Ikeji et al., 2023; Wong et al., 2022; Yoshimura et al., 2021) did not observe such findings.

Three studies (Yoshimura et al., 2018; Yoshimura et al., 2021; Yoshimura et al., 2020) examined the relationship between motor on admission and stroke-related sarcopenia. Among them, two studies (Yoshimura et al., 2018; Yoshimura et al., 2020) found that the better the motor on admission, the less likely have stroke-related sarcopenia. However, no association was found in the other study (Yoshimura et al., 2021).

Three studies (Ikeji et al., 2023; Yao et al., 2022; Kim and Choi, 2023) demonstrated that nasogastric feeding was a risk factor for stroke-related sarcopenia.

Two studies (Ikeji et al., 2023; Shimizu et al., 2022) examined the relationship between stroke types and stroke-related sarcopenia, indicating that stroke types were correlated with stroke-related sarcopenia.

The correlation between stroke duration and stroke-related sarcopenia was investigated in two studies (Aydin et al., 2021; Shimizu et al., 2022). One study (Aydin et al., 2021) discovered a significant association between stroke duration and stroke-related sarcopenia. One study (Shimizu et al., 2022) showed no differences. The other four relevant factors were reported by only one study.

#### 3.3.4 Behavioral factors

The correlation between energy adequacy (Wong et al., 2022; Shimizu et al., 2022) and stroke-related sarcopenia was investigated in two studies. One study (Wong et al., 2022) discovered a significant association between energy adequacy and stroke-related sarcopenia. One study (Shimizu et al., 2022) showed no differences.

Two studies (Mohammed and Li, 2022; Wong et al., 2022) found a significant association between smoking status and stroke-related sarcopenia, indicating that participants with a smoking history were likely to have stroke-related sarcopenia. The other three relevant factors were reported by only one study.

#### 3.3.5 Biomarkers

Two studies (Yao et al., 2022; Yoshimura et al., 2020) examined the relationship between albumin and stroke-related sarcopenia and showed that albumin was related to stroke-related sarcopenia. Two studies (Yao et al., 2022; Yoshimura et al., 2020) explored the association between hemoglobin and stroke-related sarcopenia and found a statistically significant relationship between them. The other six relevant factors were reported by only one study.

### 3.4 Clinical outcomes of stroke-related sarcopenia

#### 3.4.1 Motor outcomes

Seven studies (Yoshimura et al., 2019; Kanai et al., 2022; Matsushita et al., 2019; Abe et al., 2023; Kameyama et al., 2022; Ogino et al., 2024) explored the effect of stroke-related sarcopenia on motor function. Three studies (Yoshimura et al., 2019; Kanai et al., 2022; Arksey and O'Malley, 2005) discovered sarcopenia was independently associated with the Functional Independence Measure-motor (FIM-motor) score at discharge. However, one study (Nishioka et al., 2022) did not find the relationship. Two studies (Matsushita et al., 2019; Abe et al., 2023) revealed that sarcopenia was significantly associated with FIM-motor at discharge for men, but not for women. One study (Ogino et al., 2024) reported sarcopenia was associated with FIM-motor at discharge in the non-disability group, but not in the premorbid-disability group. The details are shown in Supplementary Table S3 and Figure 2.

#### 3.4.2 Swallowing outcomes

Two studies (Yoshimura et al., 2019; Nishioka et al., 2022) looked at the effect of stroke-related sarcopenia on swallowing outcomes, with one study (Nishioka et al., 2022) discovering sarcopenia was independently associated with a poor Food Intake Level Scale (FILS) score. And the other one (Yoshimura et al., 2019) showed no differences.

#### 3.4.3 Neurological outcomes

Five studies (Tutal et al., 2023; Jang et al., 2020; Lee et al., 2022; Lee et al., 2023; Nozoe et al., 2024) explored the effect of stroke-related sarcopenia on neurological function. Four studies (Jang et al., 2020; Lee et al., 2022; Lee et al., 2023; Nozoe et al., 2024) discovered sarcopenia was significantly associated with poor modified Rankin Scale score, and one (Jang et al., 2020) of the studies showed sarcopenia was associated with poor outcomes in men, and this association was notably stronger in women. However, one study (Tutal et al., 2023) did not find the relationship.

#### 3.4.4 Psychological outcomes

One study (Shiraishi et al., 2024) looked at the effect of stroke-related sarcopenia on depression, indicating that stroke-related sarcopenia was relevant to the Geriatric Depression Screening Scale-15 (GDS-15). One study (Ogino et al., 2024) revealed that sarcopenia was significantly associated with FIM-cognition.

#### 3.4.5 Readmission, recurrence, and mortality

One study (Abe et al., 2024) showed sarcopenia was significantly associated with readmission for stroke within 6 months. One study (Kirkham et al., 2023) showed significantly was associated with recurrent cerebrovascular events. Two studies (Tutal et al., 2023; Lee et al., 2023) looked at the effect of stroke-related sarcopenia on mortality, with one study (Tutal et al., 2023) discovering sarcopenia was independently associated with mortality. And the other one (Lee et al., 2023) showed no differences.

## 4 Discussion

This scoping review highlights the influencing factors and clinical outcomes of stroke-related sarcopenia based on 26 studies. We identified 40 relevant factors and divided them into 5 categories: demographic factors, disease factors, stroke-related factors, behavioral factors, and biomarkers. Moreover, stroke-related sarcopenia can have a profound impact on the motor, swallowing, neurological function, psychosocial health, and readmission and mortality of stroke patients. Our scoping review provided a clear mapping of the influencing factors and clinical outcomes, which can help guide research directions and intervention programs in the future.

Among multiple factors, we found that the most frequently reported risk factors for stroke-related sarcopenia were older age, lower BMI, malnutrition, severe stroke, and recurrent stroke. Unsurprisingly, older age is a risk factor for stroke-related sarcopenia. Sarcopenia is an age-related syndrome, and muscle tissue gradually decreases during the aging process, leading to a decrease in muscle mass and strength. After the age of 50, the skeletal muscle mass and muscle strength decrease at a rate of 1.0%–2.0% and 1.5%–3.0% per year, respectively. And at the age of 80, the total muscle mass and muscle strength decrease by 30% and 50%, respectively (Palus et al., 2014). Lee et al. (Lee et al., 2022) showed that the prevalence of stroke-related sarcopenia was 2.9% in patients under 50 years old and 12.0% in patients over 70 years old. Increasing BMI was significantly associated with lower odds of having stroke-related sarcopenia (Wong et al., 2022). Individuals with higher fat mass may consume lower protein, which is important for preventing muscle loss (Yu et al., 2014). So overweight may reduce the risk of sarcopenia, while obesity is a risk factor for stroke. Stroke patients need to control their weight within a reasonable range. When malnutrition occurs, there will be a lack of multiple vitamins and proteins, among which vitamin D plays a role in promoting bone growth and development and regulating calcium and phosphorus metabolism. When vitamin D is deficient, it can cause skeletal muscle atrophy (Remelli et al., 2019). Protein plays an important role in repairing tissues and cells, and participating in human material metabolism. When protein intake is insufficient, the body can only break down muscles to meet energy needs, leading to muscle relaxation (Rogeri et al., 2021). Severe stroke patients often have severe inflammation or peripheral nerve conduction disorders (Chen et al., 2022), which are associated with skeletal muscle atrophy (Nozoe et al., 2020). In addition, severe stroke is often accompanied by severe swallowing and limb dysfunction and these further exacerbate the occurrence of stroke-related sarcopenia. Recurrent stroke is usually more severe, fatal, and disabling than the first attack (Skoog and Madsen, 2022). Therefore, these patients are more likely to experience severe swallowing difficulties, hemiplegia, and cognitive impairment, which makes it easy to understand that patients with recurrent stroke are more prone to muscle atrophy.

In our review, the results of different studies differ to some extent for the same influencing factor, implying that the findings of any single study should not be overinterpreted. Future research is needed to further explore the factors impacting stroke-related sarcopenia. Moreover, interventions aimed at stroke-related sarcopenia can focus on modifiable factors, such as behavioral factors.

We found that the clinical outcomes of stroke-related sarcopenia mainly included motor, swallowing, neurological function, psychosocial health, and readmission and mortality. The diagnosis of sarcopenia is based on the decline in physical function such as skeletal muscle mass and grip strength, which is related to low activities of daily living (Wennie Huang et al., 2010). Therefore, the direct impact of poor physical function may be one of the factors that reduce motor function. Sarcopenia may also lead to secondary symptoms such as increased fatigue and reduced physical activity, further reducing physical function and affecting motor function at discharge (Fried et al., 2001). Stroke-related sarcopenia affects swallowing-related muscle groups, resulting in decreased swallowing function, reduced nutrient intake, malnutrition, and worsening muscle loss, exacerbating the process of sarcopenia (Fujishima et al., 2019). The two are a causal cycle. After a stroke, type II muscle fibers gradually degrade, resulting in a decrease in the cross-sectional area of the entire skeletal muscle. These changes in muscle volume ultimately lead to decreased mobility (Hafer-Macko et al., 2008) and affect mRS (Nozoe et al., 2018). A previous systematic review and meta-analysis have shown that there is a significant association between sarcopenia and depression (Li et al., 2022). In future clinical work, attention should be paid to screening for depression in patients with stroke-related sarcopenia, There is still a discrepant in clinical outcomes of stroke-related sarcopenia, but the scope of the impact of stroke-related sarcopenia deserves our attention. Therefore, providers should proactively carry out stroke-related sarcopenia assessment and screening, identify high-risk groups early, and conduct effective interventions to reduce the adverse outcomes associated with stroke-related sarcopenia.

## 5 Limitations

Though we developed a strict screening and search strategy among the six major databases to determine a widespread belief in results, some limitations still need to be considered. First, we only searched the English database, which may lead to publication bias due to the omission of other language literature. Second, most of the included studies were conducted in Japan, and aging and medical levels in Japan may differ from those of other countries, resulting in a higher degree of sarcopenia. Third, to provide a widespread belief, we included stroke patients of any type, age, and course of disease, and this led to a significant discrepancy in our research results. Heterogeneity in the details of the included studies made it difficult to undertake quantitative summaries across studies, leading to a lack of consistent evidence to guide clinical practice. Forth, in the studies we included, there is no report on the impact of patient rehabilitation training time and a balanced diet on stroke-related sarcopenia. Additionally, hypertension is closely associated with the occurrence of stroke and has a causal relationship with sarcopenia. These important factors need further study.

## 6 Conclusion

This scoping review summarized the influencing factors and clinical outcomes of stroke-related sarcopenia. The influencing factors for stroke-related sarcopenia include demographic, disease, stroke-related, behavioral, and biomarker factors. The most frequently reported influencing factors were older age, stroke severity, and malnutrition. Stroke-related sarcopenia can cause impaired motor, swallowing, neurological and psychological function, and lead to increased recurrence, readmission and mortality. Our findings provide a reasonably clear picture for the early identification of the population at risk of stroke-related sarcopenia. However, this review also highlighted an urgent need to unify and standardize the measurement criteria for stroke-related sarcopenia through means such as expert consensus panels. This will facilitate the systematic evaluation of clinical outcomes of stroke-related sarcopenia and enable the comparability of results across different studies. In the future, targeting these influencing factors, health professionals should develop complementary interventions to reduce adverse outcomes.
