# Supplementary material for: Stroke-related sarcopenia: a scoping review of influencing factors and clinical outcomes
Source: Front Aging. 2025 Nov 6;6:1658943. doi: 10.3389/fragi.2025.1658943 (PMC12630109; doi:10.3389/fragi.2025.1658943)
Supplement: Supplementary file 1 [file Table1.doc]

**Search Strategies（Search Date: August 13, 2024）**

| **Databases** | **Search** | **Query** | **No. of Results** |
| --- | --- | --- | --- |
| **PubMed** | #1 | "stroke"[Mesh] | 183,701 |
|  | #2 | "stroke"[Title/Abstract] OR "cerebrovascular accident"[Title/Abstract] OR "cerebrovascular disorders"[Title/Abstract] OR "cerebral infarction"[Title/Abstract] OR "cerebral hemorrhage"[Title/Abstract] | 357,962 |
|  | #3 | #1 OR #2 | 393,381 |
|  | #4 | "sarcopenia"[MeSH] | 11,188 |
|  | #5 | "sarcopenia"[Title/Abstract] OR "muscle loss"[Title/Abstract] OR "muscular atrophy"[Title/Abstract] OR "muscle weakness"[Title/Abstract] | 50,770 |
|  | #6 | #4 OR #5 | 51,784 |
|  | #7 | #3 AND #6 | ***846*** |
| **Cochrane Library** | #1 | MeSH descriptor: [stroke] explode all trees | 17,639 |
|  | #2 | (“stroke” OR “cerebrovascular accident” OR “cerebrovascular disorders” OR “cerebral infarction” OR “cerebral hemorrhage”):ti,ab,kw | 82,769 |
|  | #3 | #1 OR #2 | 82,900 |
|  | #4 | MeSH descriptor: [sarcopenia] explode all trees | 936 |
|  | #5 | (“sarcopenia” OR “muscle loss” OR “muscular atrophy” OR “muscle weakness”):ti,ab,kw | 7,475 |
|  | #6 | #4 OR #5 | 7,475 |
|  | #7 | #3 AND #6 | ***329*** |
| **Embase** | #1 | 'stroke':ab,ti,kw | 538,034 |
|  | #2 | 'sarcopenia':ab,ti,kw | 28,809 |
|  | #3 | #1 AND #2 | ***462*** |
| **CINAHL** | S1 | SU stroke | 100,525 |
|  | S2 | SU sarcopenia | 8,751 |
|  | S3 | S1 AND S2 | ***564*** |
| **Web of Science(Core Collection)** | #1 | stroke (Topic) OR "cerebrovascular accident"(Topic) OR "cerebrovascular disorders"(Topic) OR "cerebral infarction"(Topic) OR "cerebral hemorrhage"(Topic) | 495,884 |
|  | #2 | sarcopenia (Topic) OR "muscle loss"(Topic) OR "muscular atrophy"(Topic) OR "muscle weakness"(Topic) | 59,671 |
|  | #3 | #1 AND #2 | ***1,125*** |
| **Scopus** | #1 | TITLE-ABS-KEY ( stroke) | 568,688 |
|  | #2 | TITLE-ABS-KEY (sarcopenia) | 26,416 |
|  | #3 | #1 AND #2 | ***531*** |
